# Deletion of the BH3-only protein Noxa alters electrographic seizures but does not protect against hippocampal damage after status epilepticus in mice

**DOI:** 10.1038/cddis.2016.301

**Published:** 2017-01-12

**Authors:** Naoki Ichikawa, Mariana Alves, Shona Pfeiffer, Elena Langa, Yasmina E Hernández-Santana, Hidenori Suzuki, Jochen HM Prehn, Tobias Engel, David C Henshall

**Affiliations:** 1 Department of Physiology and Medical Physics, Royal College of Surgeons in Ireland, 123 St. Stephen's Green, Dublin 2, Ireland; 2 Department of Neurosurgery, Mie University Graduate School of Medicine, Tsu, Mie, Japan

## Abstract

Several members of the Bcl-2 gene family are dysregulated in human temporal lobe epilepsy and animal studies show that genetic deletion of some of these proteins influence electrographic seizure responses to chemoconvulsants and associated brain damage. The BH3-only proteins form a subgroup comprising direct activators of Bax–Bak that are potently proapoptotic and a number of weaker proapoptotic BH3-only proteins that act as sensitizers by neutralization of antiapoptotic Bcl-2 family members. Noxa was originally characterized as a weaker proapoptotic, ‘sensitizer' BH3-only protein, although recent evidence suggests it too may be potently proapoptotic. Expression of Noxa is under p53 control, a known seizure-activated pathway, although Noxa has been linked to energetic stress and autophagy. Here we characterized the response of Noxa to prolonged seizures and the phenotype of mice lacking Noxa. Status epilepticus induced by intra-amygdala kainic acid caused a rapid increase in expression of *noxa* in the damaged CA3 subfield of the hippocampus but not undamaged CA1 region. *In vivo* upregulation of *noxa* was reduced by pifithrin-*α*, suggesting transcription may be partly p53-dependent. Mice lacking *noxa* developed less severe electrographic seizures during status epilepticus in the model but, surprisingly, displayed equivalent hippocampal damage to wild-type animals. The present findings indicate Noxa does not serve as a proapoptotic BH3-only protein during seizure-induced neuronal death *in vivo*. This study extends the comprehensive phenotyping of seizure and damage responses in mice lacking specific Bcl-2 gene family members and provides further evidence that these proteins may serve roles beyond control of cell death in the brain.

Temporal lobe epilepsy (TLE) is the most common epilepsy syndrome in adults and is thought to result from an earlier injury to the brain.^1^ Repeated brief or prolonged seizures (status epilepticus (SE)) can also be directly harmful to the brain and neuron loss may contribute to the imbalances in excitation and inhibition that underlie the development and maintenance of the epileptic state.^2, 3, 4^ Current pharmacotherapy for epilepsy is symptomatic and does not alter the underlying pathophysiology.^5^ It remains a priority, therefore, to identify novel approaches to protect against seizure-induced injury to the brain.

Neuronal death caused by seizures features both necrotic and programmed/apoptotic pathways.^6, 7^ The Bcl-2 gene family encodes a large and heterogeneous group of proteins that serve important roles in promoting or opposing cell death. Members contain a variable number of up to four Bcl-2 homology (BH) domains. Among multi-BH domain members are antiapoptotic Bcl-2, Bcl-xl and Mcl-1 and proapoptotic Bax and Bak.^8, 9^ The subgroup of BH3-only proteins are separated into the potently proapoptotic members, including Bid, Bim and Puma which can directly activate Bax/Bak to promote mitochondrial release of apoptogenic proteins and the weaker BH3-only proteins that function principally by neutralizing antiapoptotic members (‘sensitizer/inactivator').^10, 11^ Expression of various members of the Bcl-2 family has been found to be altered in resected brain tissue from TLE patients and functional studies have revealed seizure and damage phenotypes for some of the multi-BH domain and potently proapoptotic members of the BH3-only subfamily.^7^ In particular, loss of Bcl-w or Mcl-1 exacerbates seizures in mice, whereas deletion of Bim or Puma partly protects the hippocampus against SE but has no effect on electrographic activity.^12, 13, 14, 15^ Notably, unexpected roles have been found in mice lacking the less potent BH3-only proteins, including increased hippocampal damage after SE in Bmf-deficient animals.^16, 17, 18^

Noxa (human gene, phorbol-12-myristate-13-acetate-induced protein 1 (*PMAIP1*/*APR*)) is a BH3-only protein discovered in a screen of p53-response genes in mouse embryonic fibroblasts subject to DNA damage.^19^ In mice, Noxa is a small protein, ~100 amino acids that localizes to mitochondria.^19^ Noxa-deficient mice are developmentally normal, although certain cells are resistant to p53-induced apoptosis.^20^ Noxa is generally placed in the proapoptotic group of sensitizer/inactivator BH3-only proteins, via targeting Mcl-1.^21^ Recent work, however, suggests Noxa also has ‘direct activator' properties.^11^ The importance of Noxa in p53-induced apoptosis has been demonstrated.^22, 23^ However, transcription factors besides p53 have been shown to induce Noxa, including RelA^24^ and FKHRL1,^25^ and non-p53-dependent roles have been described for Noxa in cell death triggered by hypoxia,^26^ stress,^27, 28^ proteasome inhibition^29^ and autophagy.^30, 31^ Noxa has also been shown to stimulate glucose consumption^32^ and been linked to nutrient stress-induced apoptosis.^33^

Noxa has potential relevance in seizure models because both p53 and Mcl-1 can influence seizure thresholds, damage and epileptogenesis.^12, 34, 35, 36^ Noxa is expressed at low levels in the brain^19^ and a variety of stressors that accompany SE activate Noxa, including axonal injury^37^ and genotoxic damage.^38^ However, studies have questioned the importance of Noxa in neuronal death induced by p53 and DNA damage^39^ and oxidative stress^40^ and in oligodendrocyte apoptosis.^41^ Here we investigated the expressional response of Noxa to SE and characterized the seizure and damage phenotype of Noxa-deficient mice.

## Results

### Noxa is selectively upregulated in hippocampal subfields after SE

As Noxa is expressed at only low levels in the normal brain, we hypothesized that if Noxa served a proapoptotic role we would observe early upregulation of *noxa* transcripts within regions of cell death after SE. To explore this idea, we analysed *noxa* levels in microdissected subfields of the mouse hippocampus after SE evoked by intra-amygdala microinjection of kainic acid (KA, 1 *μ*g).^42^ In this model, prolonged seizures are propagated from the site of injection to the hippocampus, later spreading to the neocortex.^14^ The ipsilateral cornu ammonis 3 (CA3) subfield is selectively damaged while the CA1 subfield is largely spared.^15, 17^ The dentate granule/gyrus (DG) contains a mixed population of vulnerable hilar interneurons and damage-resistant DG neurons. Cell death is evident within a few hours of SE and the lesion sites continue to expand for the next few days as cell death progresses.^42^

**Figure 1:**
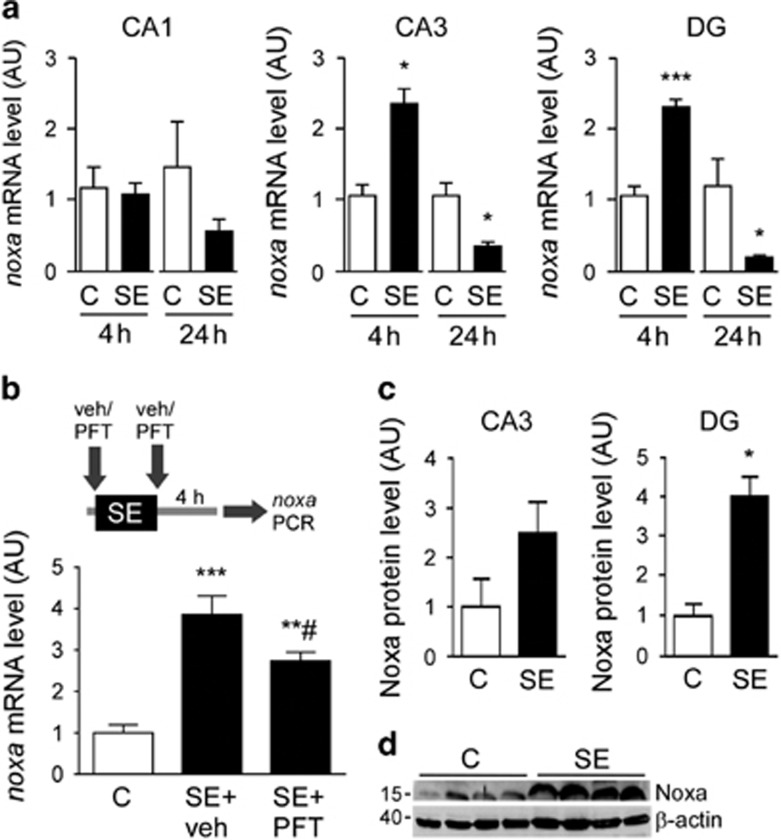
Upregulation of Noxa after SE. (**a**) Graphs show transcript data for *noxa* at 4 and 24 h in control (C) mice and in animals after status epileptics (SE) in the CA1, CA3 and DG subfields (*n*=4/group). AU, arbitrary units. (**b**) Cartoon showing experimental design in which mice were treated with a p53 inhibitor PFT (4 mg/kg) or vehicle (veh) before and after SE and *Noxa* expression was measured 4 h later. The p53 inhibitor reduced *Noxa* upregulation during SE (*n*=4/group, data from the CA3 subfield). (**c**) Graphs showing semiquantitative analysis of Noxa protein levels in the CA3 and DG subfields at 4 h and (**d**) representative western blotting for the DG (*n*=1/lane). Molecular weight markers are depicted on the left in kD. *P<0.05, **P<0.01; ***P<0.001 compared with the indicated control group; ^#^compared with SE+veh

*Noxa* expression measured using real-time quantitative PCR (RT-qPCR) was unchanged in the ipsilateral CA1 subfield at both 4 and 24 h after SE (Figure 1a). In contrast, *noxa* was increased in the CA3 subfield 4 h after SE and also in the DG subfield (Figure 1a). By 24 h after SE, expression of *noxa* was lower than controls in both the CA3 and DG subfields at 24 h (Figure 1a).

As Noxa was discovered in a screen of p53-dependent factors promoting apoptosis^19^ and the temporal profile of *noxa* upregulation followed the induction of p53 and p53-dependent genes,^15^ we explored whether *noxa* upregulation after SE required p53 transcriptional activity. Mice were injected with pifithrin-*α* (PFT), a synthetic inhibitor of the transcriptional activity of p53 previously shown to block *puma* upregulation and the p53-dependent gene *p21*^WAF^ in the same model.^15, 43^ *Noxa* levels were lower but not fully reduced in PFT-treated mice after SE compared with vehicle-treated seizure animals (Figure 1b).

To support the transcriptional data, we examined the protein levels of Noxa. Lysates from hippocampal subfields were immunoblotted using antibodies specific to Noxa (Figures 1c and d). Noxa protein was detected at very low levels in control samples, at its predicted molecular weight of ~16 kD (Figure 1d). Noxa protein levels were increased in the DG subfield at 4 h but remained unchanged in the CA1 and CA3 subfield (Figures 1c and d and data not shown). Noxa protein levels were similar to controls in all subfields at 24 h (data not shown). Noxa protein levels were not reduced by PFT treatment in mice subject to SE (Supplementary Figure S1a).

### Histological and molecular characteristics of Noxa-deficient hippocampus

**Figure 2:**
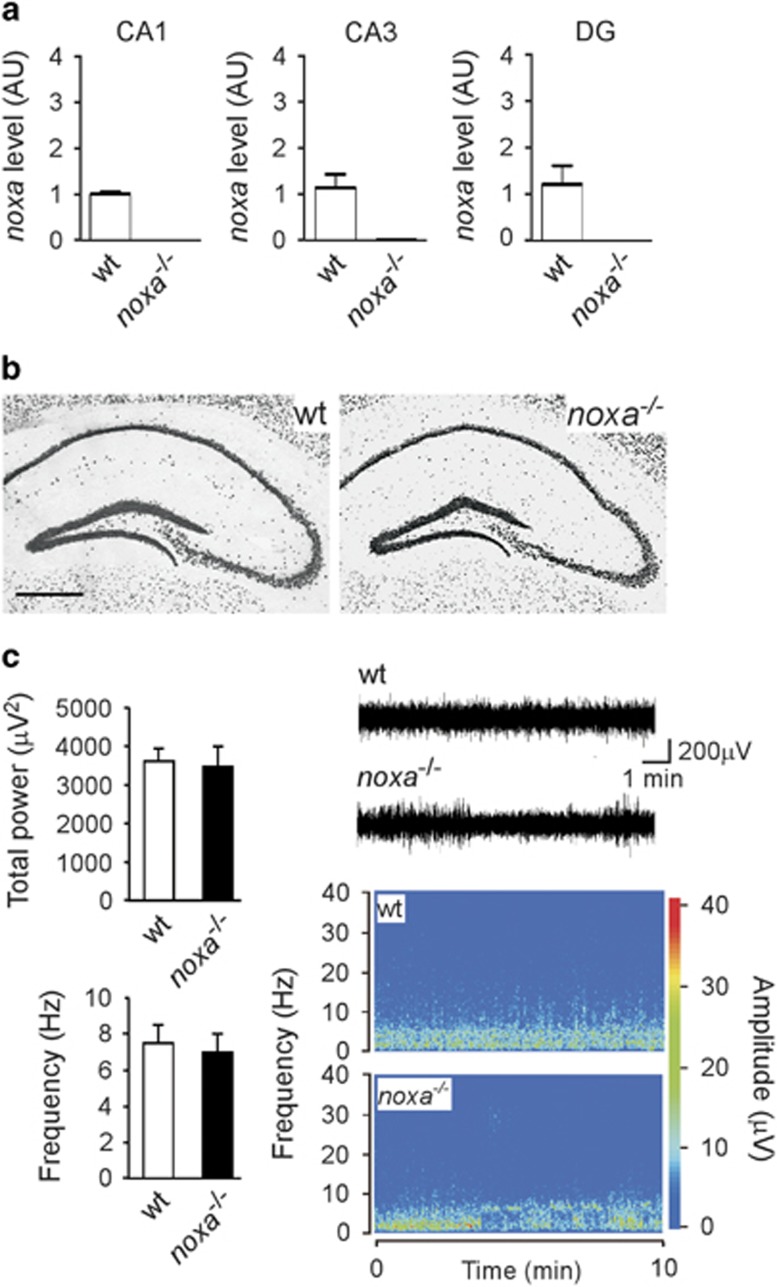
Hippocampal phenotype of mice lacking Noxa. (**a**) Real-time PCR data confirming the absence of *noxa* in Noxa-deficient mice in each of the hippocampal subfields. (**b**) Photomicrographs showing representative field views of NeuN-stained hippocampus from a control (naive) wt mouse and a section from a *noxa*^*−/−*^ animal. Note normal morphology, organization and cell density appearance of the hippocampus in Noxa-deficient mice. Scale bar, 500 *μ*m. (**c**) Graphs showing quantitative analysis of a period of baseline EEG recorded in wt and *noxa*^*−/−*^ mice (analysis performed prior to induction of SE). EEG total power and frequency data represented as a pseudocolour heat map is also included. There were no differences between genotypes (*n*=14/group). AU, arbitrary units

Noxa-deficient mice were bred normally and were born at the expected Mendelian rate, as previously reported.^20^ The absence of *noxa* was confirmed in *noxa*^*−/−*^ mice in each hippocampal subfield (Figure 2a). Nissl-stained sections of the hippocampus of *noxa*^*−/−*^ mice displayed a normal appearance, suggesting no adverse neurodevelopmental consequences of gene deletion (Figure 2b). We also compared baseline electroencephalogram (EEG) recording between *noxa*^*−/−*^ and wild-type (wt) mice (Figure 2c). No differences were found between genotypes for either resting EEG total power or frequency (Figure 2c).

### Altered electrographic seizures in Noxa-deficient mice

**Figure 3:**
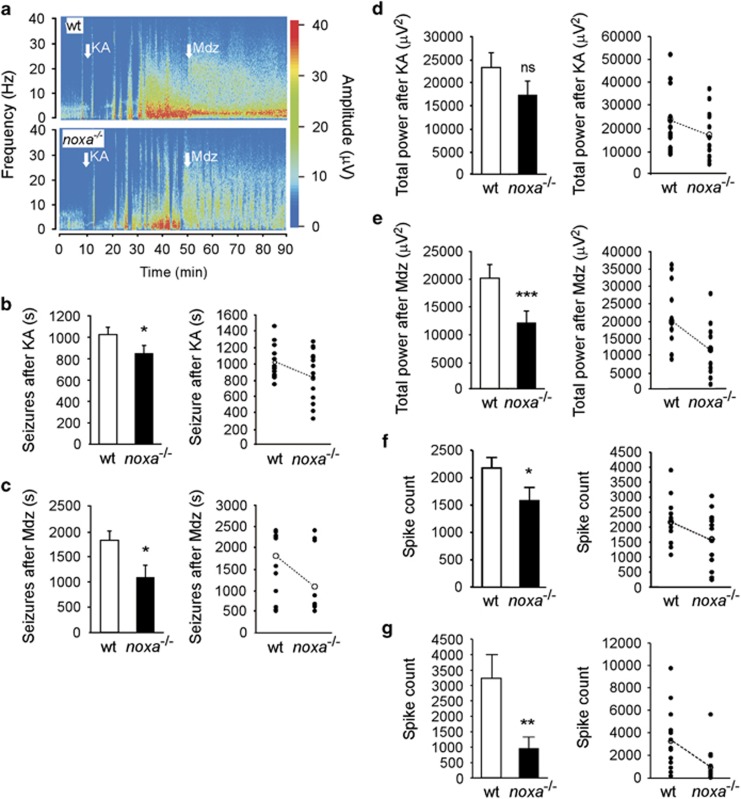
Altered electrographic seizures in mice lacking Noxa. (**a**) Representative pseudocolour heat map of EEG recorded during SE triggered by intra-amygdala KA in wt and *noxa*^*−/−*^ mice. Note slightly reduced seizure severity in *noxa*^*−/−*^ example both after KA and after injection of the anticonvulsant midazolam (Mdz; used to curtail morbidity and mortality). (**b**–**g**) Graphs showing summative data and plots of individual data from animals. Noxa-deficient mice showed (**b**, **c**) reduced seizure duration before and after midazolam. Total EEG power was not different (**d**) before KA but was different in recordings (**e**) after midazolam. Noxa-deficient mice also displayed (**f**, **g**) reduced spike counts compared with wt animals. *P<0.05; **P<0.01 compared with wt, ***P<0.001, *n*=14/group. NS, not significant

We next subjected *noxa*^*−/−*^ mice and wt animals to SE induced by intra-amygdala KA. Ink injections were performed in pilot studies to confirm correct amygdala targeting (data not shown). Intra-amygdala KA elicited SE in both *noxa*^*−/−*^ and wt mice as evidenced by the development of high amplitude, high frequency epileptiform polyspike activity on EEG (Figure 3a). Quantitative analysis of the EEG revealed *noxa*^*−/−*^ mice underwent reduced seizure severity compared with wt mice (Figures 3b–g). Manually scored seizure duration was lower in *noxa*^*−/−*^ mice during the 40 min recording period after KA injection (Figure 3b) and seizure duration was also reduced in *noxa*^*−/−*^ mice during recordings after injection of the anticonvulsant midazolam (Figure 3c), which is used in this model to curtail morbidity and mortality.^44^ Total EEG power was also lower in *noxa*^*−/−*^ mice in recordings post-midazolam (Figures 3d and e). Finally, EEG spike count, another measure of seizure activity was reduced in *noxa*^*−/−*^ mice compared with wt (Figures 3f and g). The observed seizure phenotype was not due to a dose-threshold effect because reduced seizure severity was also observed in Noxa-deficient mice when a lower dose of KA was used to elicit seizures (Supplementary Figure S2a).

### Expression of neurotransmitter signalling components in noxa^*−/−*^ mice

**Figure 4:**
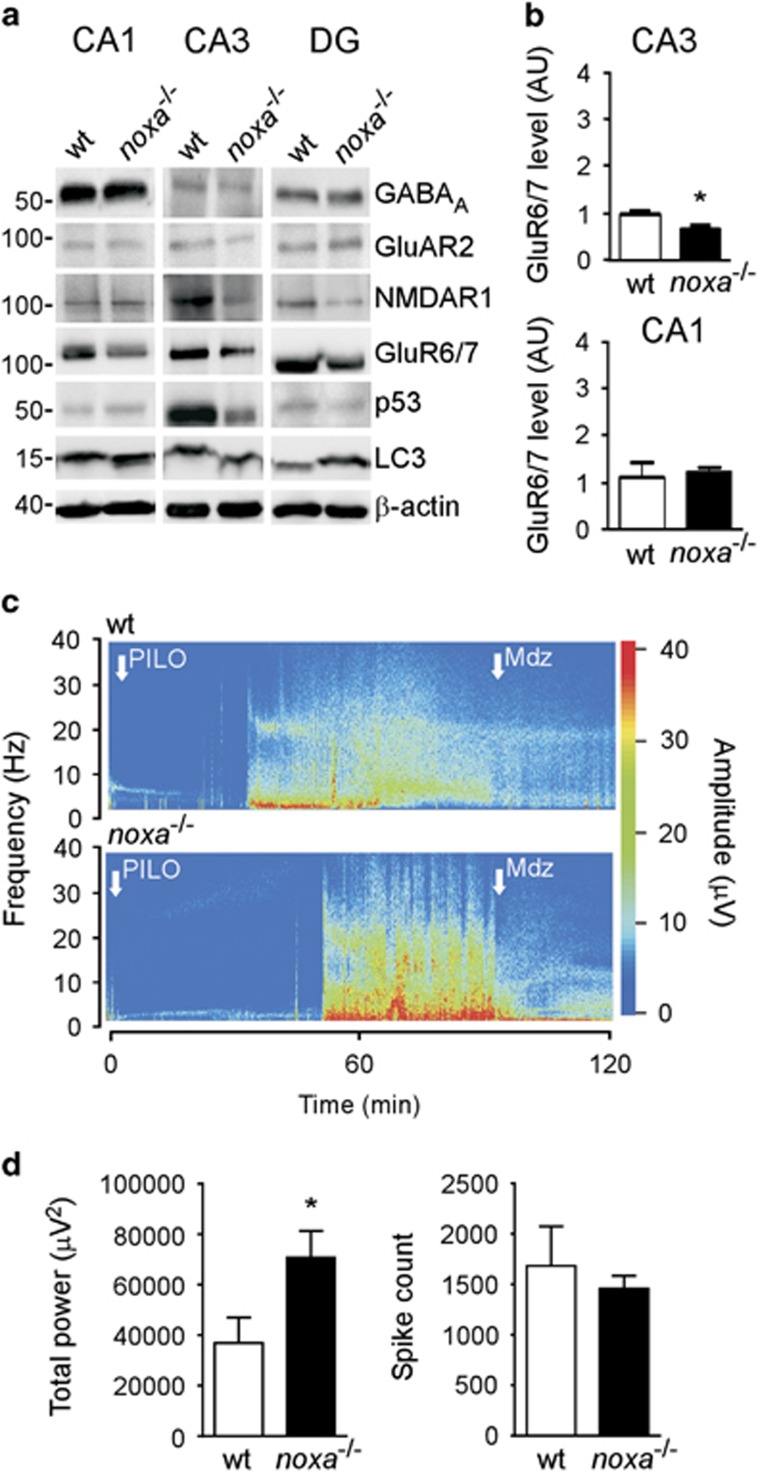
Expression of neurotransmitter receptors and response to pilocarpine in mice lacking Noxa. (**a**) Representative western blots (*n*=1/lane) showing protein levels of a selection of receptor subunits covering GABA and glutamatergic systems, including NMDA, AMPA and KA receptors, and Noxa-related signalling components p53 and LC3. Data come from the same two animals for all panels in a given subfield. Molecular weight markers are depicted on the left in kD. (**b**) Graph quantifying reduced levels of GluR6/7 in Noxa-deficient mice (*n*=4/group). (**c**) Representative pseudocolour heat maps of EEG frequency–amplitude data during SE triggered by systemic pilocarpine. (**d**) Graphs quantify EEG total power and spike count during the first 10 min of seizure activity (time-constrained owing to high mortality in *noxa*^*−/−*^ mice with this agent; *n*=5–6/group)

The altered EEG profile in *noxa*^*−/−*^ mice in the KA model was unexpected as electrographic phenotypes were not evident in previous work on four different BH3-only protein-deficient mice.^14, 15, 17, 45^ To investigate whether the altered seizures were secondary to baseline differences in neurotransmitter receptor levels in the mice, we immunoblotted proteins from *noxa*^*−/−*^ and wt mice hippocampal subfields for glutamatergic and *γ*-amino butyric acid (GABA) neurotransmitter receptor components (Figure 4a and Supplementary Figure S3). Levels of GABA_A_-R (*β*2/3) subunit were similar between *noxa*^*−/−*^ and wt mice in the three main hippocampal subfields (Figure 4a and Supplementary Figure S3). Levels of the *α*-amino-3-hydroxy-5-methyl-4-isoxazolepropionic acid (AMPA) receptor GluAR2 and the *N*-methyl-D-aspartate (NMDA)-R1 subunit was also similar in all subfields in *noxa*^*−/−*^ mice compared with wt (Figure 4a and Supplementary Figure S3). Unexpectedly, we found lower levels of the KA receptor GluR6/7 in the CA3 subfield (Figures 4a and b and Supplementary Figure S3). GluR6/7 levels in the CA1 and DG subfields of *noxa*^*−/−*^ mice were similar to wt. Moreover, treatment of mice subject to SE with PFT did not noticeably alter GluR6/7 levels (Supplementary Figure S1b). Analysis of levels of p53 and signalling components linked to Noxa-dependent autophagy (LC3 and p62) showed no difference between wt and *noxa*^*−/−*^mice (Figures 4a and data not shown).

### noxa^*−/−*^ mice are more vulnerable to pilocarpine-induced SE

Because of the different seizures in the KA model, we investigated the response of Noxa-deficient mice to a different chemoconvulsant. Here we used systemic pilocarpine, a cholinergic mimetic that is widely used to induce SE.^46^

Systemic pilocarpine triggered SE in wt and *noxa*^*−/−*^ mice (Figure 4c). Pilocarpine-induced SE was associated with significantly higher mortality in *noxa*^*−/−*^ mice (7/9 animals) compared with wt (1/6 animals) (chi-square *P*<0.05). Owing to the severe seizures and high early mortality, EEG analysis was restricted to the first 10 min of electrographic seizures after pilocarpine. This revealed that total EEG power during seizures, although not spike counts, were higher in Noxa-deficient mice compared with wt animals that was likely the cause of the increased mortality (Figures 4c and d). As with the KA model, this was not a dose-threshold effect as a similar seizure phenotype was observed in Noxa-deficient mice given a lower dose of pilocarpine to elicit seizures (Supplementary Figure S2b).

### Seizure-induced neuronal death in mice lacking Noxa

**Figure 5:**
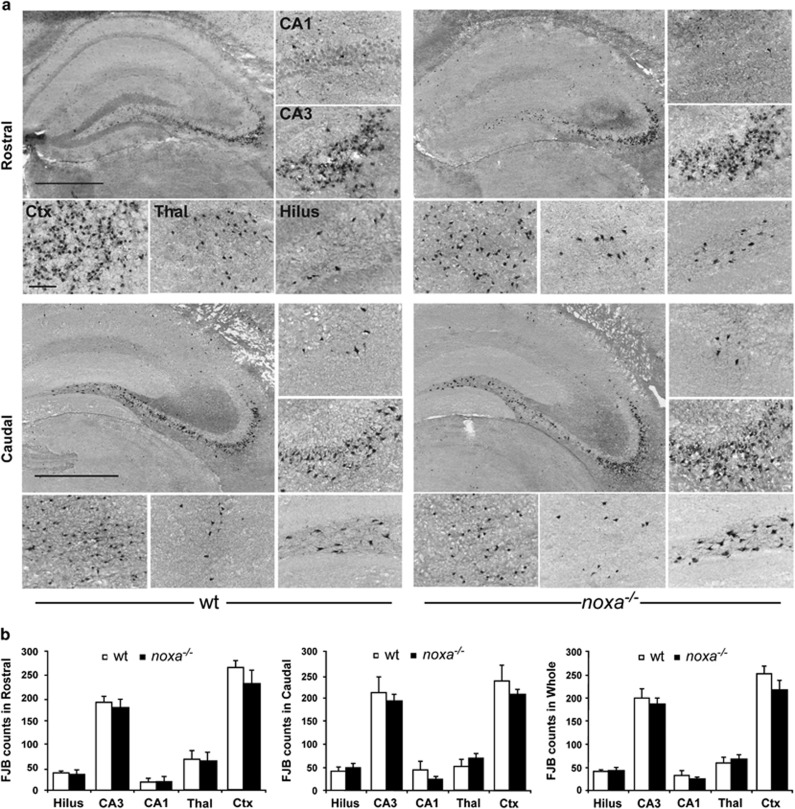
Seizure-induced neuronal death in Noxa-deficient mice. (**a**) Representative photomicrographs showing FJB staining of degenerated neurons 24 h after SE in wt and *noxa*^*−/−*^ mice at two stereotaxic levels. Seizure damage (FJB-stained cells, black dots) encompassed most of the ipsilateral CA3 subfield and hilar region of the DG, whereas only limited cell death was evidence in the CA1 subfield. Cell death was also evident in the thalamus and neocortex. Scale bars, top left, 500 *μ*m; panel below, 50 μm. (**b**) Graphs showing semiquantitative analysis of damage in wt and *noxa*^*−/−*^ mice at 24 h. There were no significant differences in neuronal death between genotypes in any subfield at either level (rostral, *n*=10/group; caudal, *n*=9; combined, *n*=19/group). Ctx, cortex; Thal, thalamus

We next investigated seizure-induced neuronal death in *noxa*^*−/−*^ mice after intra-amygdala KA. Brain tissue was not available from pilocarpine-treated mice owing to the high mortality and morbidity. Tissue sections were obtained 24 h after SE and stained using Fluorojade B (FJB), a marker of irreversible neuronal death (Figure 5). In wt mice, hippocampal sections displayed the expected pattern of damage with extensive degeneration of ipsilateral CA3 neurons (Figures 5a and b). This was evident at two different levels of the hippocampus. Damage was much less severe in the CA1 subfield and DG. There was also neurodegeneration within the neocortex and thalamus, as previously reported in this model^42^ (Figures 5a and b).

The distribution and extent of seizure-induced neuronal death was similar in *noxa*^*−/−*^ mice. The ipsilateral CA3 subfield and neocortex had the greatest number of degenerating neurons, whereas counts were lower in the CA1 and DG subfields and in the thalamus. There was no significant difference between genotypes in any examined area at either of the two stereotaxic levels (Figures 5a and b).

Given that *noxa*^*−/−*^ mice had less severe seizures during SE in the KA model, the equivalent hippocampal damage could indicate that *noxa*^*−/−*^ mice are more vulnerable to seizure-induced neuronal death. That is, they experienced a less severe insult but developed comparable damage. To explore this idea, we assessed damage in a subgroup of mice in which seizures were comparable. Analysis of rostral and caudal CA3 damage in a subgroup of *noxa*^*−/−*^ mice with similar seizure durations to wt mice found no significant difference between groups (Supplementary Figure S4). Thus, even when adjusted for seizure severity, *noxa*^*−/−*^ mice do not display altered neuronal death in this model.

**Figure 6:**
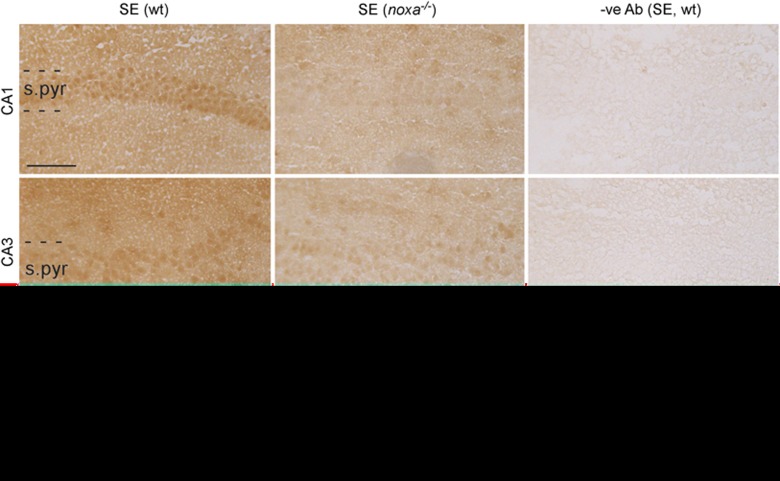
Noxa immunostaining in seizure-damaged hippocampi from wt and Noxa-deficient mice. Representative photomicrographs showing Noxa immunostaining in the main hippocampal subfields 24 h after SE. Noxa immunoreactivity is mainly confined to neuronal populations in wt mice (SE (wt)). There was minimal Noxa immunoreactivity in mice lacking *noxa* (SE (*noxa*^*−/−*^)). Noxa immunoreactivity was completely eliminated in tissue sections from wt mice in which the primary antibody was omitted (−Ab (SE, wt)). Scale bar, 100 *μ*m. s.pyr, stratum pyramidale; gcl, granule cell layer

Finally, we supported our histological analysis by staining tissue sections using antibodies specific to Noxa. Noxa immunoreactivity was detectable in the CA1, CA3 and hilus/DG subfields of the ipsilateral hippocampus of wt mice subject to SE (Figure 6). Immunoreactivity was mainly localized to the neuronal cell bodies. In contrast, Noxa immunoreactivity was minimal in hippocampal subfields of *noxa*^*−/−*^ mice subject to SE (Figure 6). Noxa immunoreactivity was absent in tissue sections from wt mice subject to SE in which the primary antibody was omitted (Figure 6).

## Discussion

Understanding the molecular contributors to seizure-induced neuronal death may offer important insights to neuroprotection as activation of cell death pathways contributes to lesion development that precipitates epilepsy.^2, 3, 4^ The present study brings closer to completion the genetic determination of the contribution, or lack thereof, of Bcl-2 family proteins to seizure-induced neuronal death.^7^ Although canonical proapoptotic roles have been found for two of the three potently proapoptotic BH3-only proteins, unexpected damage and seizure phenotypes have been reported for some of the weakly proapoptotic BH3-only proteins. The present study expands these insights into the roles of BH3-only proteins in seizure-induced neuronal death and reveals unexpected seizure phenotypes in *noxa*^*−/−*^ mice. Noxa is interesting because it regulates cell death in various tissues in response to stressors that are triggered by SE, including DNA damage, oxidative stress and nutrient deprivation.^47, 48^ Noxa has previously been linked to control of neuronal and glial apoptosis,^37, 38^ but the present study is the first to explore a functional role for Noxa in seizure-induced neuronal death. Also, it recently emerged that Noxa may be a BH3-only protein with ‘direct activator' properties^11^ and that Noxa is also constitutively expressed in the brain, albeit at low levels.^19^ A major finding in the present study is that Noxa does not have a proapoptotic role during seizure-induced neuronal death *in vivo*. In contrast to the protection seen in mice lacking BH3-only proteins Puma and Bim,^14, 15^ seizure damage was the same as wt levels in mice lacking Noxa. We evaluated damage in five different brain areas, making this the most comprehensive assessment of cell death after seizures in a mouse lacking a Bcl-2 family member. Thus Noxa, along with Bid,^45^ is not important for seizure-induced neuronal death. These data agree with findings in other models where Noxa was not required for neuronal death.^39, 40^ Further studies will be required to determine whether introduction or overexpression of Noxa can alter seizure-induced neuronal death or exert other effects in hippocampal neurons.

In the present study, we found early induction of Noxa in hippocampal subfields that develop damage after SE in this model. The time course was similar to that found for other BH3-only proteins, including Puma, which is p53 dependent in the same model.^14, 15^ Noxa induction was earlier than Bmf, upregulation of which is AMPK dependent in the same model.^17^ The upregulation of Noxa is also faster than the response of neurons to a pure DNA-damaging agent,^39^ suggesting other stimuli may drive Noxa induction after seizures. A previous study that investigated *noxa* levels after SE did not find transcriptional changes.^15^ Changes to *noxa* expression could have been missed in that study because of the use of whole hippocampus. This emphasises the importance of the subfield-specific analyses performed presently. Surprisingly, *noxa* levels dropped below control levels at 24 h. This is unusual for a BH3-only protein in this model and may be unique to Noxa. It is possible that this reflects early degeneration of neurons although this time point is still relatively early and the response was comparable between DG and CA3 despite substantial differences in the extent of neuronal death between these areas. Notably, SE produces select changes to DNA methylation in this model^49^ and epigenetic silencing of the *noxa* gene has been reported.^50^ Another possibility is that expression is reduced posttranscriptionally, for example, by a microRNA.^51^

The transcriptional control of *noxa* was originally found to be p53 dependent.^19^ We focussed on p53 here because it has previously been found to control seizure-induced neuronal death and is upregulated in refractory human TLE.^34, 35, 36^ PFT was used to block p53 transcriptional activity using a well-established dosing regime,^15^ and we found p53 inhibition resulted in a small reduction in *noxa* expression after SE. These data suggest that p53 has a role in the control of *noxa* levels in this model. The incomplete suppression of *noxa* by PFT and lack of effect on Noxa protein suggests that other transcription factors or mechanisms also control *noxa* levels,^24, 25^ although identification of these lies outside the scope of the present study.

Analysis of baseline EEG parameters did not find any difference between wt and Noxa-deficient mice, supporting a lack of gross differences in brain function at rest consistent with the macroscopic appearance of the brains. During SE, however, we found that seizure duration and spiking triggered by intra-amygdala KA was reduced in mice lacking Noxa. This was not related to a threshold effect as reduced seizures also occurred in *noxa*^*−/−*^ mice when a lower dose of KA was injected. Reductions in seizure time and total power were also evident during recordings after midazolam.^44^ Taken together, these findings suggest that Noxa may have a role in promoting brain excitability or the development of synchronous neuronal firing. A proexcitability phenotype would be unusual for a Bcl-2 family member. Indeed, electrographic seizure responses to intra-amygdala KA are normal for mice lacking Bim,^14^ Bid,^45^ Puma^15^ and Bmf^17^ and multi-BH domain proapoptotic Bok.^52^ Seizures were exacerbated in mice lacking multi-BH domain antiapoptotic members, including Mcl-1^12^ and Bcl-w.^13^ The only BH3-only protein mutants known to display reduced seizures are *bad*^*−/−*^ mice.^16^ One potential mechanism underlying the reduced seizures in Noxa-deficient mice is lower levels of ionotropic glutamate receptors, which were observed in the CA3. No such regulation of glutamatergic receptor components has previously been reported in studies of seizures in mice lacking either constitutively expressed or transcriptionally upregulated members of the Bcl-2 family, suggesting that this may be unique to *noxa*^*−/−*^ mice. Whether this difference is important is uncertain. It is unlikely that the small reduction in GluR6/7 levels accounts for the electrographic seizure phenotype. Although the precise contribution of glutamatergic signalling to seizure activity in the intra-amygdala KA model is unknown, the EEG signal is likely dominated by AMPA receptor-driven epileptiform activity.^53, 54^ Furthermore, *noxa*^*−/−*^ mice were more vulnerable to pilocarpine, an agent that triggers seizures through a different transmitter system. This was an unexpected finding. Indeed, previous work characterizing seizure phenotypes in mice lacking Bcl-2 family members found similar results between models.^16^ However, divergence in response between KA and pilocarpine models has been reported for other targets.^55, 56^ The difference between KA and pilocarpine responses here may relate to the mechanism and signalling pathways by which seizures are triggered in the two models. Although intra-amygdala KA triggers seizures via activation of glutamatergic signalling pathways, systemic pilocarpine-induced SE involves changes to peripheral immune cells and blood–brain barrier disruption.^57^ Notably, increased vulnerability to pilocarpine was also reported for mice lacking the Noxa target Mcl-1^12^ and the genes show substantial overlapping expression in the mouse brain. A more comprehensive analysis of neurotransmitter expression may be warranted to resolve the present findings. Regardless, the present study supports a potential novel role for Noxa in modifying synchronous, high-intensity neuronal firing behaviour *in vivo* extending its known roles beyond the control of cell death in the brain.

The present study used the same model of focal-onset SE as used previously in assessments of other Bcl-2 family proteins. This ensures we can interpret the findings without the problem of the model as a source of variability. Nevertheless, a number of limitations should be considered. As we used constitutive knockout mice we cannot exclude that the altered chemoconvulsant responses in Noxa-deficient mice are a consequence of an effect on neurodevelopment. Indeed, Noxa has been linked to the control of neural precursor cell death.^38^ Experiments using heterozygous (*noxa*^*+/−*^ mice) animals would provide useful insights on dose-dependent effects at the genetic level. It would also be interesting to investigate whether the p53 inhibitor affects seizures or pathological outcomes in Noxa-deficient mice. Although Noxa immunoreactivity appeared mainly neuronal, we did not explicitly identify the cell type(s) in which Noxa was expressed. Noxa has been linked to both glial and neuronal death^37, 38^ and both processes may occur in the present model. We did not explore the targets of Noxa such as Mcl-1, which serves an antiapoptotic role in seizure-induced neuronal death.^12^ In summary, the present study identifies *noxa* as a transcriptionally responsive gene to SE and shows that genetic deletion of this gene leads to altered seizure severity in models of SE. As with several other members of the Bcl-2 family, Noxa's proapoptotic role is not evident during seizure-induced neuronal death *in vivo*, supporting roles for this protein beyond control of cell death.

## Materials and Methods

### Breeding of wt and noxa^−/−^ mice

Targeted *noxa* mutant mice were provided by Professor Andreas Strasser (WEHI, Melbourne, Australia). They were originally generated from C57BL/6-derived Bruce4 ES cells backcrossed onto a C57Bl/6J background for >10 generations and mutant and wt littermates were bred as homozygous colonies.^20, 58^ Genotyping was performed as described.^58^

### Focal-onset SE in mice

Animal procedures were performed in accordance with the principals of the European Communities Council Directive (2010/63/EU) and were reviewed and approved by the Research Ethics Committee of the Royal College of Surgeons in Ireland, under licenses from the Health Products Regulatory Authority, Ireland. Studies were performed according to previously described techniques.^15, 17^ Adult male mice (20–25 g) (C57BL/6 (Harlan, Blackthorn, UK), wt and *noxa* ^*−/−*^ mice underwent SE induced by unilateral sterotaxic microinjection of KA (Sigma-Aldrich, Ireland Limited, Arklow, Ireland) into the basolateral amygdala nucleus. Briefly, mice were anesthetized using isoflurane (2–5%) and maintained normothermic by means of a feedback-controlled heat blanket (Harvard Apparatus Ltd, Kent, England). Next mice were placed in a stereotaxic frame, and following a midline scalp incision, three partial craniotomies were performed. Three recording electrodes (Bilaney Consultants Ltd, Sevenoaks, UK) were then affixed to the skull. EEG was recorded using a Grass Comet digital EEG (Medivent Ltd, Lucan, Ireland). A guide cannula was affixed over the dura (coordinates from Bregma: AP=−0.94; L=−2.85 mm) and the entire skull assembly was fixed in place with dental cement. Anaesthesia was discontinued, EEG recordings were commenced, and then a 31-gauge internal cannula (Bilaney Consultants Ltd) was inserted into the lumen of the guide to inject KA (1 or 0.1 *μ*g/0.2 *μ*l of vehicle; phosphate-buffered saline (PBS), pH adjusted to 7.4) into the amygdala. Non-seizure control animals received the same volume of intra-amygdala vehicle. The EEG was recorded until intraperitoneal midazolam (8 mg/kg) administration at 40 min.^44^ Mice were killed after 4 or 24 h after anticonvulsant, and the brains were microdissected on ice or flash-frozen whole in 2-methylbutane at −30 °C for histopathology. Brains from additional naive (non-instrumented) wt and *noxa*^*−/−*^ mice were used to examine hippocampal neuroanatomy and basal gene expression.

### Pilocarpine-induced SE

Pilocarpine (340 or 300 mg/kg intraperitoneal, Sigma-Aldrich Ireland, Dublin, Ireland) was injected into additional *noxa*^*−/−*^ or wt mice 20 min after methyl-scopolamine (1 mg/kg; given to prevent peripheral cholinergic side effects) to trigger SE, as described.^46^

### EEG analysis

Digitized EEG recordings were analysed off-line using manual assessment and automated software, as described.^15, 17^ The duration of high-frequency (>5 Hz) and high-amplitude (>2 × baseline) polyspike discharges of ⩾5 s duration, which are synonymous with injury-causing electrographic activity, was counted by a reviewer blind to treatment. Automated EEG analysis was performed by uploading EEG into the Labchart7 software (ADInstruments, Oxford, UK) to calculate total power and spike counts from the EEG signal. EEG recordings were separated into the 40 min period after intra-amygdala KA injection up to the time of anticonvulsant administration and a second epoch covering a period of 40 min after anticonvulsant.

### p53 inhibitor treatment

C57BL/6 mice received injections of the p53 transcriptional inhibitor PFT (1-(4-methylphenyl)-2-(4,5,6,7-tetrahydro-2-imino-3(2H)-benzothiazolyl)-ethanone hydrobromide; Santa Cruz Biotechnology, Santa Cruz, CA, USA).^15^ Seizure mice received intraperitoneal injection of either vehicle (PBS) or PFT (4 mg/kg) 24 h before and 1 h after the induction of SE. We have previously reported that this PFT dosing regimen does not alter severity of SE.^15^ Animals were killed 4 h after midazolam for analysis of *noxa* expression.

### Western blotting

Western blotting was performed as previously.^15, 17^ Hippocampal subfields were homogenized in lysis buffer and protein concentration was determined. In all, 30 *μ*g protein samples were then boiled in gel-loading buffer and separated on 10–15% SDS-PAGE gels. Proteins were transferred onto nitrocellulose membranes (Bio-Rad, Hemel Hempstead, UK) and incubated with antibodies against the following: GluAR2 and NMDAR1 (Antibodies Inc., Davis, CA, USA), GluR6/7 (Millipore, Tullagreen, Ireland), GABA-A receptor *β*2/3 subunit (Millipore), LC3 and p62 (Abgent, San Diego, CA, USA), Noxa (Abcam, Cambridge, UK), p53, *α*-Tubulin (Santa Cruz Biotechnology), *β*-Actin (Sigma-Aldrich) and GAPDH (Cell Signaling Technology, Danvers, MA, USA). Membranes were then incubated with horseradish peroxidase-conjugated secondary antibodies (Isis Ltd, Kildare, Ireland) and bands were visualized using Supersignal West Pico Chemiluminescent Substrate (Thermofisher Scientific, Waltham, MA, USA). Images were captured using a Fuji-film LAS-300 (Fuji, Sheffield, UK), densitometry was performed using the AlphaEaseFC4.0 software (Alpha Innotech, San Leandaro, CA, USA) and data were expressed as change relative to control.

### RNA extraction and RT-qPCR

RNA was extracted using Trizol (Thermofisher Scientific) protocol as described.^15, 17^ Briefly, 1 *μ*g total RNA was used to generate cDNA by reverse transcription using Superscript II Reverse Transcriptase enzyme (Thermofisher Scientific). Quantitative real-time PCR was performed using a LightCycler 1.5 (Roche Diagnostics, Indianapolis, IN, USA) in combination with the QuantiTech SYBR Green PCR Kit (Qiagen Ltd, Manchester, UK) as per the manufacturers' protocol and 1.25 *μ*M of primer pair was used. Data were analysed by the LightCycler 1.5 software; data were normalized to the expression of *β*-Actin and represented at RQ values. Primers were designed using the Primer3 software (http://frodo.wi.mit.edu) and verified by BLAST (http://blast.ncbi.nlm.nih.gov/Blast.cgi). Primer sequences of *noxa* were: 5′-TCAGGAAGATCGGAGACAAA-3′ and 5′-TGAGCACACTCGTCCTTCAA-3′.

### Histopathology

Neuronal damage was assessed using FJB (Millipore), as described.^15, 17^ Fresh-frozen coronal brain tissue sections (12 *μ*m) were postfixed in formalin, treated with 0.006% potassium permanganate solution, rinsed and transferred to FJB solution (0.001% in 0.1% acetic acid) (Millipore). After staining, sections were rinsed again, dried, cleared and mounted in DPX (Sigma-Aldrich). Sections were imaged using a LEICA DM4000B epifluorescence microscope with LEICA DFC 310FX camera (Laboratory Instruments & Supplies (I) Ltd, Ashbourne, Ireland). Fluorescence images were converted to greyscale and inverted such that degenerating neurons appeared black on a light grey background. Semiquantification of damaged cells was performed at two levels of the hippocampus for the CA1, CA3 and DG subfields, the thalamus and neocortex. Counts were the average of two adjacent sections assessed by an observer masked to experimental group/condition.

Noxa immunostaining was performed as previously described^46^ using a mouse monoclonal antibody specifically recommended for immunohistochemistry (Cat. no. 200-301-H98, Rockland Immunochemicals Inc, Limerick, PA, USA). Briefly, tissue sections from wt and *noxa*^*−/−*^ mice not used for FJB staining were fixed and blocked followed by incubation with the primary antibody (1:100 dilution). Non-specific staining was assessed by omission of the primary antibody. Tissue sections were rinsed and then incubated with secondary antibodies, and immunostaining was visualized using the standard HRP-diaminobenzidine staining (Vector Laboratories Ltd, Peterborough, UK).^46^ Images of the staining were taken using equal exposure times on a Leica DM 4000B microscope and no changes to contrast or brightness were applied.

### Data analysis

Data are presented as means±S.E.M. Data were analysed using ANOVA with *post hoc* Fisher's PLSD test and Student's *t*-test for two-group comparisons (StatView software; SAS Institute, Cary, NC, USA). Significance was accepted at *P*<0.05.

## Abbreviations

AMPA: *α*-amino-3-hydroxy-5-methyl-4-isoxazolepropionic acid
BH: Bcl-2 homology
CA3: cornu ammonis 3
DG: dentate granule/gyrus
EEG: electroencephalogram
FJB: Fluorojade B
GABA: *γ*-amino butyric acid
KA: kainic acid
NMDA: *N*-methyl-D-aspartate
PBS: phosphate-buffered saline
PFT: pifithrin-*α*
RT-qPCR: real-time quantitative PCR
SE: status epilepticus
TLE: temporal lobe epilepsy
wt: wild type

## References

[bib1] Cendes F, Sakamoto AC, Spreafico R, Bingaman W, Becker AJ. Epilepsies associated with hippocampal sclerosis. Acta Neuropath 2014; 128: 21–37. 24823761 10.1007/s00401-014-1292-0

[bib2] Sloviter RS. Status epilepticus-induced neuronal injury and network reorganization. Epilepsia 1999; 40(Suppl 1): S34–S39. 10421559 10.1111/j.1528-1157.1999.tb00876.x

[bib3] Henshall DC, Meldrum BS. Cell death and survival mechanisms after single and repeated brief seizures. Jasper's Basic Mechanisms of the Epilepsies, 4th edn. Oxford University Press: Bethesda, MD, USA, 2012, pp 262–276.

[bib4] Dingledine R, Varvel NH, Dudek FE. When and how do seizures kill neurons, and is cell death relevant to epileptogenesis? Adv Exp Med Biol 2014; 813: 109–122. 25012371 10.1007/978-94-017-8914-1_9 PMC4624106

[bib5] Loscher W, Klitgaard H, Twyman RE, Schmidt D. New avenues for anti-epileptic drug discovery and development. Nat Rev Drug Disc 2013; 12: 757–776. 10.1038/nrd4126 24052047

[bib6] Fujikawa DG. Neuroprotective strategies in status epilepticus. In: Wasterlain CG, Treiman DM (eds). Status Epilepticus: Mechanisms and management vol. 36. MIT Press: Cambridge, MA, USA, 2006, pp 463–480.

[bib7] Henshall DC, Engel T. Contribution of apoptosis-associated signaling pathways to epileptogenesis: lessons from Bcl-2 family knockouts. Front Cell Neurosci 2013; 7: 110. 23882182 10.3389/fncel.2013.00110 PMC3712126

[bib8] Youle RJ, Strasser A. The BCL-2 protein family: opposing activities that mediate cell death. Nat Rev Mol Cell Biol 2008; 9: 47–59. 18097445 10.1038/nrm2308

[bib9] Chipuk JE, Moldoveanu T, Llambi F, Parsons MJ, Green DR. The BCL-2 family reunion. Mol Cell 2010; 37: 299–310. 20159550 10.1016/j.molcel.2010.01.025 PMC3222298

[bib10] Kim H, Rafiuddin-Shah M, Tu HC, Jeffers JR, Zambetti GP, Hsieh JJ et al. Hierarchical regulation of mitochondrion-dependent apoptosis by BCL-2 subfamilies. Nat Cell Biol 2006; 8: 1348–1358. 17115033 10.1038/ncb1499

[bib11] Chen HC, Kanai M, Inoue-Yamauchi A, Tu HC, Huang Y, Ren D et al. An interconnected hierarchical model of cell death regulation by the BCL-2 family. Nat Cell Biol 2015; 17: 1270–1281. 26344567 10.1038/ncb3236 PMC4589531

[bib12] Mori M, Burgess DL, Gefrides LA, Foreman PJ, Opferman JT, Korsmeyer SJ et al. Expression of apoptosis inhibitor protein Mcl1 linked to neuroprotection in CNS neurons. Cell Death Differ 2004; 11: 1223–1233. 15286683 10.1038/sj.cdd.4401483

[bib13] Murphy B, Dunleavy M, Shinoda S, Schindler C, Meller R, Bellver-Estelles C et al. Bcl-w protects hippocampus during experimental status epilepticus. Am J Pathol 2007; 171: 1258–1268. 17702891 10.2353/ajpath.2007.070269 PMC1988875

[bib14] Murphy BM, Engel T, Paucard A, Hatazaki S, Mouri G, Tanaka K et al. Contrasting patterns of Bim induction and neuroprotection in Bim-deficient mice between hippocampus and neocortex after status epilepticus. Cell Death Differ 2010; 17: 459–468. 19779495 10.1038/cdd.2009.134 PMC2950266

[bib15] Engel T, Murphy BM, Hatazaki S, Jimenez-Mateos EM, Concannon CG, Woods I et al. Reduced hippocampal damage and epileptic seizures after status epilepticus in mice lacking proapoptotic Puma. FASEB J 2010; 24: 853–861. 19890018 10.1096/fj.09-145870 PMC3231945

[bib16] Gimenez-Cassina A, Martinez-Francois JR, Fisher JK, Szlyk B, Polak K, Wiwczar J et al. BAD-dependent regulation of fuel metabolism and K(ATP) channel activity confers resistance to epileptic seizures. Neuron 2012; 74: 719–730. 22632729 10.1016/j.neuron.2012.03.032 PMC3361694

[bib17] Moran C, Sanz-Rodriguez A, Jimenez-Pacheco A, Martinez-Villareal J, McKiernan RC, Jimenez-Mateos E et al. Bmf upregulation through the AMP-activated protein kinase pathway may protect the brain from seizure-induced cell death. Cell Death Dis 2013; 4: e606. 23618904 10.1038/cddis.2013.136 PMC3668628

[bib18] Hardwick JM, Soane L. Multiple functions of BCL-2 family proteins. Cold Spring Harbor Persp Biol 2013; 5: a008722. 10.1101/cshperspect.a008722 PMC3552500 23378584

[bib19] Oda E, Ohki R, Murasawa H, Nemoto J, Shibue T, Yamashita T et al. Noxa, a BH3-only member of the Bcl-2 family and candidate mediator of p53-induced apoptosis. Science 2000; 288: 1053–1058. 10807576 10.1126/science.288.5468.1053

[bib20] Villunger A, Michalak EM, Coultas L, Mullauer F, Bock G, Ausserlechner MJ et al. p53- and drug-induced apoptotic responses mediated by BH3-only proteins puma and noxa. Science 2003; 302: 1036–1038. 14500851 10.1126/science.1090072

[bib21] Ploner C, Kofler R, Villunger A. Noxa: at the tip of the balance between life and death. Oncogene 2008; 27(Suppl 1): S84–S92. 19641509 10.1038/onc.2009.46 PMC3272398

[bib22] Schuler M, Maurer U, Goldstein JC, Breitenbucher F, Hoffarth S, Waterhouse NJ et al. p53 triggers apoptosis in oncogene-expressing fibroblasts by the induction of Noxa and mitochondrial Bax translocation. Cell Death Differ 2003; 10: 451–460. 12719722 10.1038/sj.cdd.4401180

[bib23] Michalak EM, Villunger A, Adams JM, Strasser A. In several cell types tumour suppressor p53 induces apoptosis largely via Puma but Noxa can contribute. Cell Death Differ 2008; 15: 1019–1029. 18259198 10.1038/cdd.2008.16 PMC2974267

[bib24] Inta I, Paxian S, Maegele I, Zhang W, Pizzi M, Spano P et al. Bim and Noxa are candidates to mediate the deleterious effect of the NF-kappa B subunit RelA in cerebral ischemia. J Neurosci 2006; 26: 12896–12903. 17167080 10.1523/JNEUROSCI.3670-06.2006 PMC6674970

[bib25] Obexer P, Geiger K, Ambros PF, Meister B, Ausserlechner MJ. FKHRL1-mediated expression of Noxa and Bim induces apoptosis via the mitochondria in neuroblastoma cells. Cell Death Differ 2007; 14: 534–547. 16888645 10.1038/sj.cdd.4402017

[bib26] Kim JY, Ahn HJ, Ryu JH, Suk K, Park JH. BH3-only protein Noxa is a mediator of hypoxic cell death induced by hypoxia-inducible factor 1alpha. J Exp Med 2004; 199: 113–124. 14699081 10.1084/jem.20030613 PMC1887730

[bib27] Li J, Lee B, Lee AS. Endoplasmic reticulum stress-induced apoptosis: multiple pathways and activation of p53-up-regulated modulator of apoptosis (PUMA) and NOXA by p53. J Biol Chem 2006; 281: 7260–7270. 16407291 10.1074/jbc.M509868200

[bib28] Zhang L, Lopez H, George NM, Liu X, Pang X, Luo X. Selective involvement of BH3-only proteins and differential targets of Noxa in diverse apoptotic pathways. Cell Death Differ 2011; 18: 864–873. 21113147 10.1038/cdd.2010.152 PMC3074052

[bib29] Craxton A, Butterworth M, Harper N, Fairall L, Schwabe J, Ciechanover A et al. NOXA, a sensor of proteasome integrity, is degraded by 26S proteasomes by an ubiquitin-independent pathway that is blocked by MCL-1. Cell Death Differ 2012; 19: 1424–1434. 22361683 10.1038/cdd.2012.16 PMC3422467

[bib30] Abedin MJ, Wang D, McDonnell MA, Lehmann U, Kelekar A. Autophagy delays apoptotic death in breast cancer cells following DNA damage. Cell Death Differ 2007; 14: 500–510. 16990848 10.1038/sj.cdd.4402039

[bib31] Elgendy M, Sheridan C, Brumatti G, Martin SJ. Oncogenic Ras-induced expression of Noxa and Beclin-1 promotes autophagic cell death and limits clonogenic survival. Mol Cell 2011; 42: 23–35. 21353614 10.1016/j.molcel.2011.02.009

[bib32] Lowman XH, McDonnell MA, Kosloske A, Odumade OA, Jenness C, Karim CB et al. The proapoptotic function of Noxa in human leukemia cells is regulated by the kinase Cdk5 and by glucose. Mol Cell 2010; 40: 823–833. 21145489 10.1016/j.molcel.2010.11.035

[bib33] Wensveen FM, Alves NL, Derks IA, Reedquist KA, Eldering E. Apoptosis induced by overall metabolic stress converges on the Bcl-2 family proteins Noxa and Mcl-1. Apoptosis 2011; 16: 708–721. 21516346 10.1007/s10495-011-0599-8 PMC3098366

[bib34] Morrison RS, Wenzel HJ, Kinoshita Y, Robbins CA, Donehower LA, Schwartzkroin PA. Loss of the p53 tumor suppressor gene protects neurons from kainate- induced cell death. J Neurosci 1996; 16: 1337–1345. 8778285 10.1523/JNEUROSCI.16-04-01337.1996 PMC6578556

[bib35] Engel T, Murphy BM, Schindler CK, Henshall DC. Elevated p53 and lower MDM2 expression in hippocampus from patients with intractable temporal lobe epilepsy. Epilepsy Res 2007; 77: 151–156. 17942278 10.1016/j.eplepsyres.2007.09.001 PMC2204088

[bib36] Engel T, Tanaka K, Jimenez-Mateos EM, Caballero-Caballero A, Prehn JH, Henshall DC. Loss of p53 results in protracted electrographic seizures and development of an aggravated epileptic phenotype following status epilepticus. Cell Death Dis 2010; 1: e79. 21368852 10.1038/cddis.2010.55 PMC3035899

[bib37] Kiryu-Seo S, Hirayama T, Kato R, Kiyama H. Noxa is a critical mediator of p53-dependent motor neuron death after nerve injury in adult mouse. J Neurosci 2005; 25: 1442–1447. 15703398 10.1523/JNEUROSCI.4041-04.2005 PMC6726006

[bib38] Akhtar RS, Geng Y, Klocke BJ, Latham CB, Villunger A, Michalak EM et al. BH3-only proapoptotic Bcl-2 family members Noxa and Puma mediate neural precursor cell death. J Neurosci 2006; 26: 7257–7264. 16822983 10.1523/JNEUROSCI.0196-06.2006 PMC6673947

[bib39] Wyttenbach A, Tolkovsky AM. The BH3-only protein Puma is both necessary and sufficient for neuronal apoptosis induced by DNA damage in sympathetic neurons. J Neurochem 2006; 96: 1213–1226. 16478523 10.1111/j.1471-4159.2005.03676.x

[bib40] Steckley D, Karajgikar M, Dale LB, Fuerth B, Swan P, Drummond-Main C et al. Puma is a dominant regulator of oxidative stress induced Bax activation and neuronal apoptosis. J Neurosci 2007; 27: 12989–12999. 18032672 10.1523/JNEUROSCI.3400-07.2007 PMC6673275

[bib41] Hagemeier K, Lurbke A, Hucke S, Albrecht S, Preisner A, Klassen E et al. Puma, but not noxa is essential for oligodendroglial cell death. Glia 2013; 61: 1712–1723. 23922240 10.1002/glia.22552

[bib42] Mouri G, Jimenez-Mateos E, Engel T, Dunleavy M, Hatazaki S, Paucard A et al. Unilateral hippocampal CA3-predominant damage and short latency epileptogenesis after intra-amygdala microinjection of kainic acid in mice. Brain Res 2008; 1213: 140–151. 18455706 10.1016/j.brainres.2008.03.061

[bib43] Culmsee C, Zhu X, Yu QS, Chan SL, Camandola S, Guo Z et al. A synthetic inhibitor of p53 protects neurons against death induced by ischemic and excitotoxic insults, and amyloid beta-peptide. J Neurochem 2001; 77: 220–228. 11279278 10.1046/j.1471-4159.2001.t01-1-00220.x

[bib44] Diviney M, Reynolds JP, Henshall DC. Comparison of short-term effects of midazolam and lorazepam in the intra-amygdala kainic acid model of status epilepticus in mice. Epilepsy Behav 2015; 51: 191–198. 26291773 10.1016/j.yebeh.2015.07.038

[bib45] Engel T, Caballero-Caballero A, Schindler CK, Plesnila N, Strasser A, Prehn JH et al. BH3-only protein Bid is dispensable for seizure-induced neuronal death and the associated nuclear accumulation of apoptosis-inducing factor. J Neurochem 2010; 115: 92–101. 20646170 10.1111/j.1471-4159.2010.06909.x

[bib46] Engel T, Sanz-Rodgriguez A, Jimenez-Mateos EM, Concannon CG, Jimenez-Pacheco A, Moran C et al. CHOP regulates the p53-MDM2 axis and is required for neuronal survival after seizures. Brain 2013; 136: 577–592. 23361066 10.1093/brain/aws337

[bib47] Wasterlain CG, Fujikawa DG, Penix L, Sankar R. Pathophysiological mechanisms of brain damage from status epilepticus. Epilepsia 1993; 34(Suppl 1): S37–S53. 8385002 10.1111/j.1528-1157.1993.tb05905.x

[bib48] Danial NN, Hartman AL, Stafstrom CE, Thio LL. How does the ketogenic diet work? Four potential mechanisms. J Child Neurol 2013; 28: 1027–1033. 23670253 10.1177/0883073813487598 PMC3971996

[bib49] Miller-Delaney SF, Das S, Sano T, Jimenez-Mateos EM, Bryan K, Buckley PG et al. Differential DNA methylation patterns define status epilepticus and epileptic tolerance. J Neurosci 2012; 32: 1577–1588. 22302800 10.1523/JNEUROSCI.5180-11.2012 PMC6703365

[bib50] Yamashita M, Kuwahara M, Suzuki A, Hirahara K, Shinnaksu R, Hosokawa H et al. Bmi1 regulates memory CD4 T cell survival via repression of the Noxa gene. J Exp Med 2008; 205: 1109–1120. 18411339 10.1084/jem.20072000 PMC2373843

[bib51] Lerner M, Haneklaus M, Harada M, Grander D. MiR-200c regulates Noxa expression and sensitivity to proteasomal inhibitors. PLoS One 2012; 7: e36490. 22615771 10.1371/journal.pone.0036490 PMC3352905

[bib52] D'Orsi B, Engel T, Pfeiffer S, Nandi S, Kaufmann T, Henshall DC et al. Bok is not pro-apoptotic but suppresses PARP-dependent cell death pathways and protects against excitotoxic and seizure-induced neuronal injury. J Neurosci 2016; 36: 4564–4578. 27098698 10.1523/JNEUROSCI.3780-15.2016 PMC6601822

[bib53] Young D, Dragunow M. Non-NMDA glutamate receptors are involved in the maintenance of status epilepticus. Neuroreport 1993; 5: 81–83. 8280865 10.1097/00001756-199310000-00022

[bib54] Castro-Alamancos MA, Borrell J. Contribution of NMDA and nonNMDA glutamate receptors to synchronized excitation and cortical output in the primary motor cortex of the rat. Brain Res Bull 1995; 37: 539–543. 7633903 10.1016/0361-9230(95)00059-n

[bib55] Kim JE, Kang TC. The P2X7 receptor-pannexin-1 complex decreases muscarinic acetylcholine receptor-mediated seizure susceptibility in mice. J Clin Invest 2011; 121: 2037–2047. 21505260 10.1172/JCI44818 PMC3083785

[bib56] Engel T, Gomez-Villafuertes R, Tanaka K, Mesuret G, Sanz-Rodriguez A, Garcia-Huerta P et al. Seizure suppression and neuroprotection by targeting the purinergic P2X7 receptor during status epilepticus in mice. FASEB J 2012; 26: 1616–1628. 22198387 10.1096/fj.11-196089

[bib57] Marchi N, Oby E, Batra A, Uva L, De Curtis M, Hernandez N et al. *In vivo* and *in vitro* effects of pilocarpine: relevance to ictogenesis. Epilepsia 2007; 48: 1934–1946. 17645533 10.1111/j.1528-1167.2007.01185.x PMC3900294

[bib58] Pfeiffer S, Anilkumar U, Chen G, Ramirez-Peinado S, Galindo-Moreno J, Munoz-Pinedo C et al. Analysis of BH3-only proteins upregulated in response to oxygen/glucose deprivation in cortical neurons identifies Bmf but not Noxa as potential mediator of neuronal injury. Cell Death Dis 2014; 5: e1456. 25299781 10.1038/cddis.2014.426 PMC4237251

