# Supplementary material for: Deletion of the BH3-only protein Noxa alters electrographic seizures but does not protect against hippocampal damage after status epilepticus in mice
Source: Cell Death Dis. 2017 Jan 12;8(1):e2556–. doi: 10.1038/cddis.2016.301 (PMC5457684; doi:10.1038/cddis.2016.301)
Supplement: Supplementary Figure S1 [file cddis2016301x1.pdf]

Supplementary data Figure S1

Western blot analysis of the effects of p53 inhibitor PFT on protein levels of Noxa and GluR6/7 after status epilepticus in wild-type mice

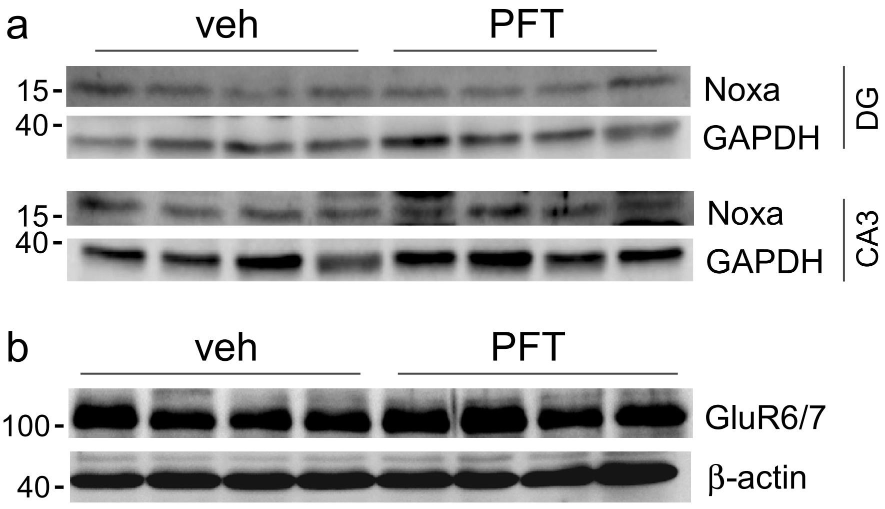

Figure S1.

Representative western blots (*n* = 1/lane) showing protein levels in hippocampal samples from wild-type mice treated with PFT or vehicle at the time of KA-induced status epilepticus or (a) Noxa and (b) GluR6/7, which had been found to be differentially expressed in naive Noxa-deficient mice. Molecular weight markers depicted on left in kD. DG, dentate gyrus.
