# Supplementary material for: Deletion of the BH3-only protein Noxa alters electrographic seizures but does not protect against hippocampal damage after status epilepticus in mice
Source: Cell Death Dis. 2017 Jan 12;8(1):e2556–. doi: 10.1038/cddis.2016.301 (PMC5457684; doi:10.1038/cddis.2016.301)
Supplement: Supplementary Figure S2 [file cddis2016301x2.pdf]

Supplementary data Figure S2

Electrographic seizure parameters in wild-type and $noxa^{-/-}$ mice in lower-dose KA and pilocarpine models

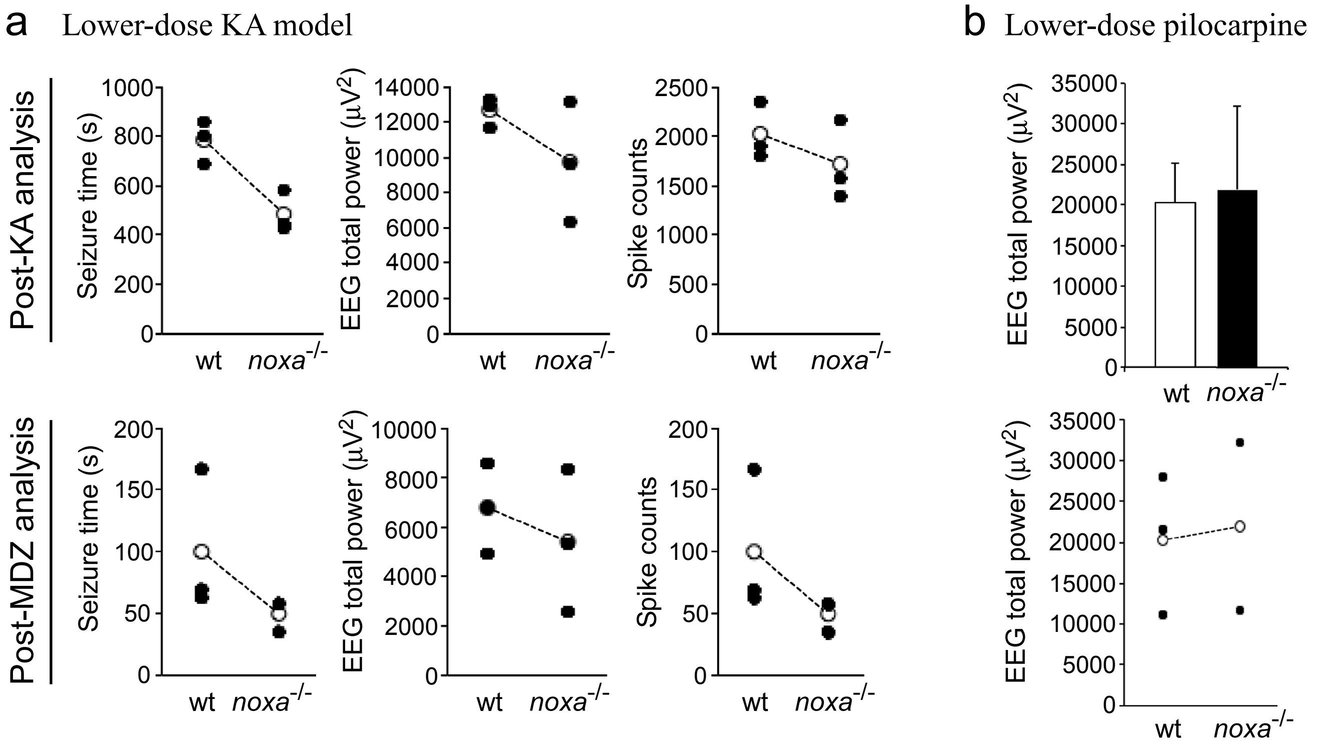

Figure S2.

(a) Analysis of electrographic seizure parameters in a lower dose KA model. Seizures were induced by intraamygdala microinjection of 0.1 μg KA in wild-type (wt) and Noxa-deficient (*$noxa^{-/-}$*) mice. Seizure parameters were quantified for the time between KA injection and 40 min (post-KA analysis) and for the time after midazolam (MDZ) injection (Post-MDZ analysis). Noxa-deficient mice consistently showed reduced seizure parameters in the low-dose KA model compared to wild-type animals. *n* = 3/group. White circles and dashed lines show group average data.

(b) Analysis of electrographic seizure parameters in a lower dose pilocarpine model. Status epilepticus was induced by 300 mg/kg (i.p.) pilocarpine in wild-type and Noxa-deficient mice. Graphs showing summative data and plots of individual data from animals. The more limited EEG analysis was due to high mortality in the model which limited the amount of available data that could be analyzed. *n* = 2-3/group.
