# Supplementary material for: Deletion of the BH3-only protein Noxa alters electrographic seizures but does not protect against hippocampal damage after status epilepticus in mice
Source: Cell Death Dis. 2017 Jan 12;8(1):e2556–. doi: 10.1038/cddis.2016.301 (PMC5457684; doi:10.1038/cddis.2016.301)
Supplement: Supplementary Figure S3 [file cddis2016301x3.pdf]

Supplementary data Figure S3

Additional immunoblots for different proteins associated with neurotransmission and Noxa-related signaling in naive wild-type and noxa$^{-/-}$ mice

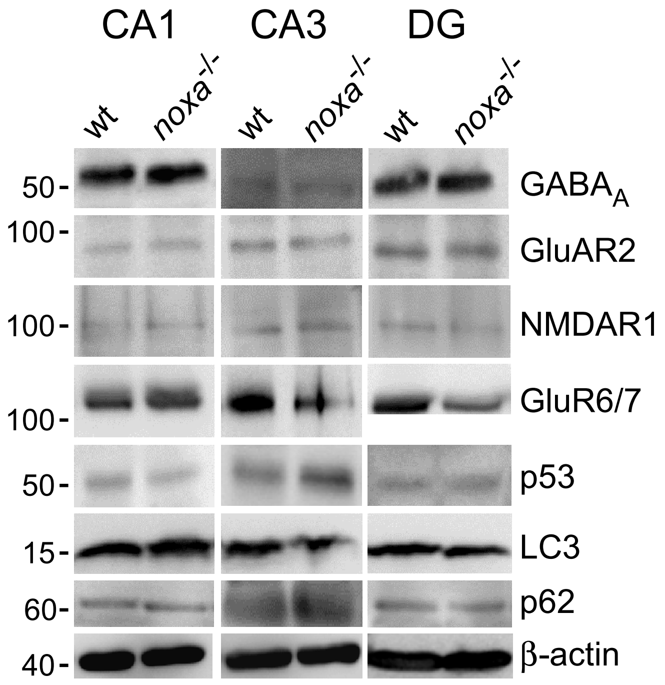

Figure S3.
Additional representative western blots (n = 1/lane) showing protein levels in naive brain samples of a selection of neurotransmitter receptor subunits covering GABA and glutamatergic systems, including NMDA, AMPA and KA receptors as well as Noxa-related signalling components p53, p62 and LC3. Note similar proteins levels between wild-type (wt) and Noxa-deficient mice (*noxa*$^{-/-}$) for each subfield with the exception of lower levels of GluR6/7 in the CA3 subfield of *noxa*$^{-/-}$ mice. Molecular weight markers depicted on left in kD.
