# Supplementary material for: Deletion of the BH3-only protein Noxa alters electrographic seizures but does not protect against hippocampal damage after status epilepticus in mice
Source: Cell Death Dis. 2017 Jan 12;8(1):e2556–. doi: 10.1038/cddis.2016.301 (PMC5457684; doi:10.1038/cddis.2016.301)
Supplement: Supplementary Figure S4 [file cddis2016301x4.pdf]

Supplementary data Figure S4

Seizure-induced hippocampal damage in noxa$^{-/-}$ mice with equivalent seizure duration to wild-type animals

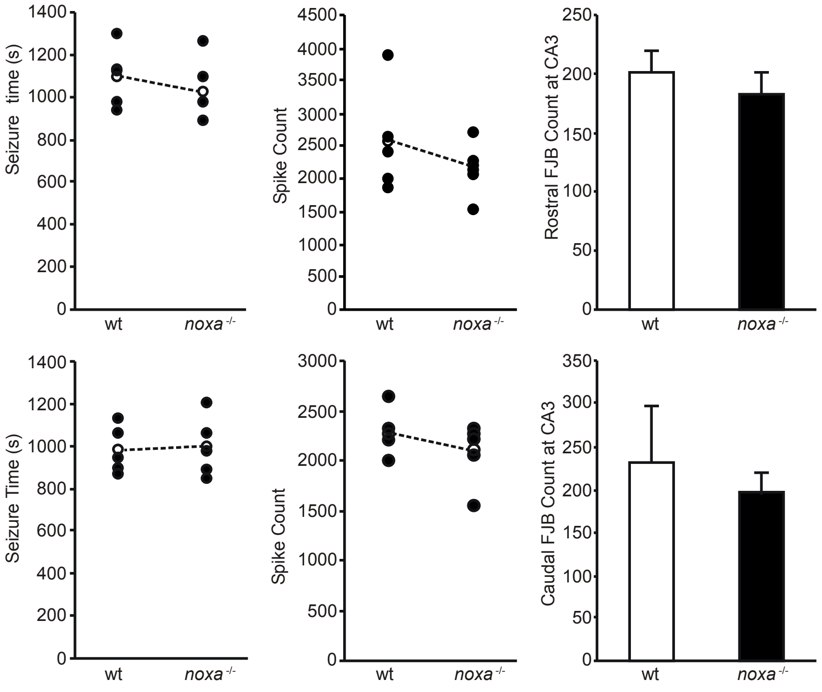

Figure S4. Sub-group analysis of hippocampal CA3 damage in *noxa*$^{-/-}$ mice with similar seizure parameters to wild-type (wt) mice. A selection of mice with similar seizure durations and spike counts are shown along with the FJB counts at rostral and caudal levels of hippocampus. Note that when differences in seizures are minimized in this way there is still no difference in damage between groups.
